# BCR Affinity Influences T-B Interactions and B Cell Development in Secondary Lymphoid Organs

**DOI:** 10.3389/fimmu.2021.703918

**Published:** 2021-07-26

**Authors:** Alec J. Wishnie, Tzippora Chwat-Edelstein, Mary Attaway, Bao Q. Vuong

**Affiliations:** ^1^ Biology PhD Program, Graduate Center, The City University of New York, New York, NY, United States; ^2^ Department of Biology, The City College of New York, New York, NY, United States; ^3^ Macaulay Honors College, New York, NY, United States

**Keywords:** BCR affinity, T follicular helper cells, germinal center, extrafollicular, B cell

## Abstract

B cells produce high-affinity immunoglobulins (Igs), or antibodies, to eliminate foreign pathogens. Mature, naïve B cells expressing an antigen-specific cell surface Ig, or B cell receptor (BCR), are directed toward either an extrafollicular (EF) or germinal center (GC) response upon antigen binding. B cell interactions with CD4^+^ pre-T follicular helper (pre-Tfh) cells at the T-B border and effector Tfh cells in the B cell follicle and GC control B cell development in response to antigen. Here, we review recent studies demonstrating the role of B cell receptor (BCR) affinity in modulating T-B interactions and the subsequent differentiation of B cells in the EF and GC response. Overall, these studies demonstrate that B cells expressing high affinity BCRs preferentially differentiate into antibody secreting cells (ASCs) while those expressing low affinity BCRs undergo further affinity maturation or differentiate into memory B cells (MBCs).

## Introduction

B cells mediate the humoral immune response through the production of antigen-specific immunoglobulins (Igs) that neutralize foreign pathogens (1). After developing in the bone marrow from hematopoietic stem cells, B cells express a plasma membrane bound Ig, termed the B cell receptor (BCR), and localize to secondary lymphoid organs (SLOs), such as the spleen and lymph nodes (1, 2). B cells form B cell follicles within SLOs, where they first encounter soluble antigen or antigen presented by professional antigen presenting cells (APCs) (3, 4). In the B cell follicle, antigen and T cells stimulate B cells to alter the Ig genes. B cells change the constant region of the Ig heavy chains through class switch recombination (CSR), which alters the expressed Ig isotype from IgM to IgG, IgE, or IgA (1, 5). Unlike CSR, somatic hypermutation (SHM) generates mutations within the variable region of the Ig light and heavy chains to promote affinity maturation (5, 6). Both CSR and SHM require the enzyme activation induced cytidine deaminase (AID), as inactivating mutations in AID in mice and humans completely block both processes (5, 7). Interestingly, AID deficiency also increases the size of germinal centers and the number of germinal center B cells (5, 7).

Prior to the induction of CSR and SHM, antigen-binding to the naïve BCR induces B cell migration to the border of the T cell zone and the B cell follicle (T-B border) (8, 9). B cells migrate to the T-B border by upregulating the chemokine receptor CCR7, which responds to the T cell zone chemokines CCL19 and CCL21. These B cells also maintain expression of the chemokine receptor CXCR5, which responds to the B cell follicle chemokine CXCL13 to prevent entry into the T cell zone (9). At the T-B border, B cells interact with pre-T follicular helper (Tfh) cells, a type of CD4^+^ T helper (Th) cell, for the first time (8, 10). Here, BCR-antigen affinity influences the interactions between B cells and pre-Tfh cells and directs B cells toward either an extrafollicular (EF) or germinal center (GC) response (11–15). Both responses promote development and differentiation of B cells into memory B cells (MBCs); short-lived, highly proliferative plasmablasts (PBs); or terminally differentiated plasma cells (PCs) with varying lifespans (1, 16, 17). The EF response occurs earlier and results in lower affinity Igs than the GC response (18). Additionally, the EF response produces shorter lived MBCs and PCs, whereas the GC response generates longer lived MBCs and PCs (3).

The mechanisms that regulate mature B cell development in the SLOs remain unclear, specifically regarding differentiation, migration within SLOs, and the location for CSR and SHM. In this review, we discuss the signals controlling B cell progression through the EF or GC response, emphasizing the role of T-B interactions and BCR affinity in B cell fate determination. We also present emerging theories on the temporal regulation of Ig diversification within the SLO.

## BCR Affinity and T Cell Help Direct B Cells to an EF or GC Response

**Figure 1:**
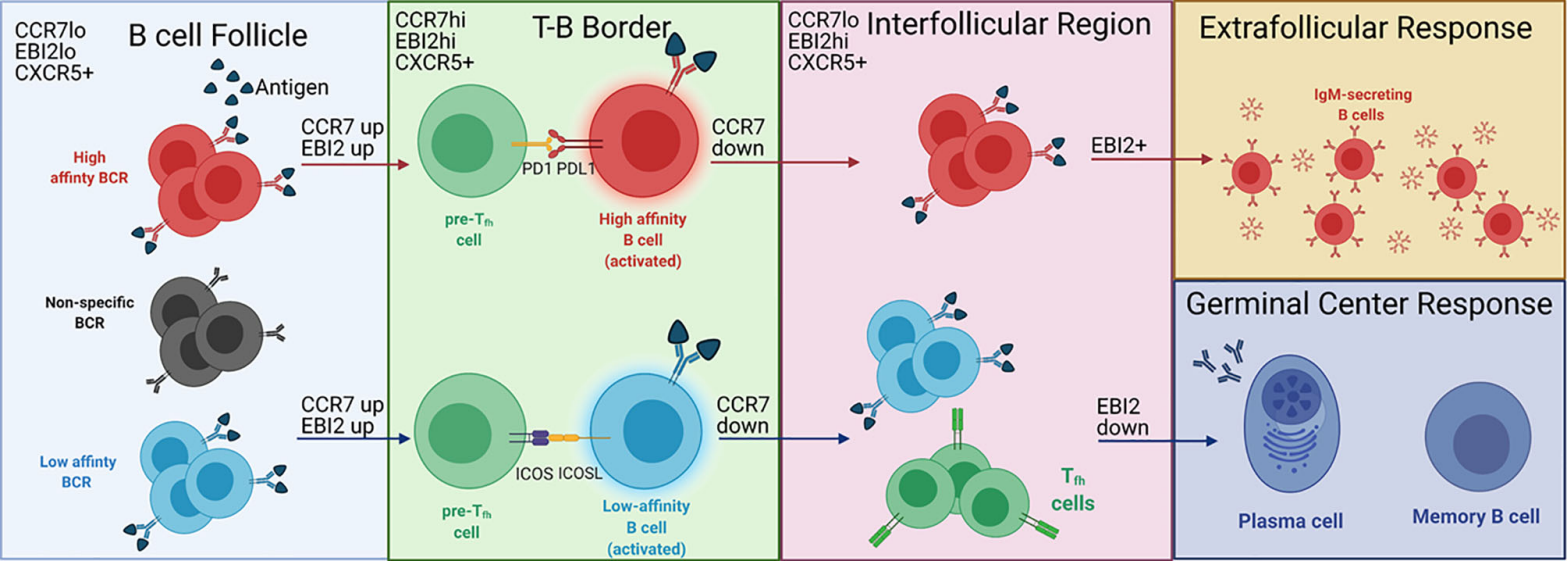
BCR affinity controls EF versus GC B cell development at the T-B border. Antigen binding naïve B cells in the B cell follicle migrate to the T-B border after upregulating CCR7 and EBI2. B cells that do not bind antigen (black) cannot migrate to the T-B border. At the T-B border, B cells with high affinity BCRs (red cells) are PDL1^hi^, allowing for PDL1-PD1 interactions with pre-Tfh cells (green cells). B cells expressing low affinity BCRs (blue cells) show stronger ICOS-ICOSL interactions with pre-Tfh cells, which induces Tfh differentiation (green cells expressing TCR in the interfollicular region). After interacting with pre-Tfh cells at the T-B border, B cells downregulate CCR7 and migrate to the interfollicular region. B cells with high affinity BCRs maintain EBI2 expression and participate in the EF response. B cells with low affinity BCRs downregulate EBI2 and enter the GC, leading to the production of long-lived plasma cells and memory B cells. The labels on the top left of each region depict the B cell expression profile of chemokine receptors that regulate localization to the respective region. This is not specified for the EF and GC responses as the expression of the chemokine receptors for each response is unclear. Adapted from reference (13).

After binding antigen, B cells undergo an EF or GC response, which depends in part on the BCR affinity for its antigen (3, 19) (**Figure 1** **)**. Higher affinity BCRs preferentially induce an EF response, while lower affinity BCRs preferentially induce a GC response (11, 14, 15). BCR affinity also influences T-B cell interactions at the T-B border which, in turn, direct B cells to form a GC in the follicle or an EF response in the EF foci in the bridging channels of the spleen or medulla in the lymph nodes (20, 21).

BCR binding to antigen induces expression of B cell ligands that bind to receptors on the pre-Tfh cell surface (20). Naïve B cells exposed to high doses of α-IgM antibody, which crosslinks the BCR and mimics high affinity antigen binding to the BCR, significantly downregulate inducible T cell costimulator ligand (ICOSL) and upregulate programmed death ligand 1 (PDL1) *in vitro* (20). Ligation of ICOSL to ICOS on pre-Tfh cells promotes differentiation of the pre-Tfh cells into effector Tfh cells (22–25). Conversely, ligation of PDL1 to PD1 on pre-Tfh cells inhibits Tfh differentiation (26–29). Thus, naïve B cells expressing low affinity BCRs, which are destined for a GC response, promote differentiation of pre-Tfh cells into effector Tfh cells, whereas B cells expressing high affinity BCRs inhibit Tfh differentiation for a Tfh-independent EF response. Surprisingly, immunization of MD4 transgenic mice with high affinity hen egg lysozyme (HEL) or low affinity duck egg lysozyme (DEL) does not recapitulate the downregulation of ICOSL observed following *in vitro* stimulation of BCR with high levels of α-IgM (20). However, inhibiting ICOS-ICOSL interactions with a α-ICOSL antibody in MD4 mice immunized with DEL, but not HEL, prevents Tfh differentiation *in vivo*, suggesting that naïve B cells expressing low affinity BCRs promote Tfh differentiation *in vivo* through ICOSL (20). This result also demonstrates a role for antigen-specific B cells in the differentiation of pre-Tfh into Tfh cells, which contradicts a previous hypothesis that bystander B cells provide the only source of ICOSL involved in Tfh differentiation (20, 25). Bystander B cells are not well characterized, but do not bind antigen and constitutively express ICOSL (25). The role of bystander B cells in Tfh differentiation may explain the maintenance of ICOSL by high affinity B cells *in vivo*. Potentially, high affinity B cells express ICOSL to prevent pre-Tfh cells from interacting with bystander B cells. The upregulation of PDL1 by high affinity B cells may provide sufficient inhibitory signals to prevent ICOS-induced Tfh differentiation (20). However, PDL1 expression in relation to BCR affinity at the T-B border has not been evaluated, indicating that further studies are needed to understand the regulation of this ligand *in vivo*.

In addition to influencing the direct interactions between pre-Tfh and B cells, BCR affinity impacts B cell localization to the sites of the EF and GC responses by altering the expression of chemokine receptors on the B cell surface as demonstrated by Sacquin and colleagues (20). Within 24 hours of BCR stimulation, at which point B cells should be localized to the T-B border, B cells stimulated with α-IgM upregulate CCR7. Higher affinity B cells, as represented by increased BCR stimulation through α-IgM, upregulate CCR7 to a higher degree than lower affinity B cells. This results in a higher ratio of CCR7:CXCR5 in high affinity B cells destined for an EF response compared to low affinity B cells destined for a GC response. Because CCR7 promotes migration toward the T cell zone while CXCR5 promotes migration toward the B cell follicle, the higher CCR7:CXCR5 ratio induced by high affinity BCRs should maintain B cells near the follicular periphery, away from the center follicle where the GC develops. Thus, these data provide a mechanism by which BCR affinity controls B cell development by directing B cell localization in the follicle (9, 30).

EBI2, another chemokine receptor, also directs B cells toward an EF or GC response. The EBI2 ligand (EBI2L) is 7α,25-dihydroxycholesterol (7α,25-OHC), which is present in the interfollicular (IF) regions but absent from the GC (31, 32). Generally, EBI2 is associated with localization to the outer follicle, suggesting that it isolates B cells from the GC, possibly in conjunction with CXCR4 (33–35). Additionally, along with CXCR5 and downregulation of CCR7, EBI2 directs B cell migration from the T-B border to the IF region (**Figure 1** **)** (19, 32). Within the IF region, B cells destined for an EF response maintain EBI2 expression while those destined for the GC downregulate EBI2 (35). However, the signals that induce downregulation of EBI2 are unknown. ICOS-ICOSL interactions could be a signal involved in EBI2 downregulation, as EF T cell help to B cells is ICOSL independent while GC T cell help is ICOSL dependent (13, 36). Further evaluation of ICOS-ICOSL interactions in the IF region could help elucidate the regulatory signals required for B cell development in this region.

Expression of B cell lymphoma 6 protein (BCL6) is a key indicator of the GC response and another B cell intrinsic protein whose expression is influenced by BCR affinity (37, 38). Upon initial antigen encounter, B cells modulate their expression of BCL6 through the activity of interferon regulatory factor 4 (IRF4) (38, 39). High affinity B cells repress BCL6 by expressing higher levels of IRF4 (37, 40). As BCL6 expression is imperative to the GC response, the increase in IRF4 expression promotes an EF response through repression of BCL6 (37). Conversely, low affinity B cells activate lower levels of IRF4 and, in this context, IRF4 has an activating effect on BCL6, thereby promoting a GC response (37). Whether IRF4 activates or represses BCL6 depends on the region of the BCL6 locus that it binds (37). Overall, the inter-relationship between BCR affinity, IRF4, and BCL6 demonstrates how BCR affinity influences B cell development upon initial antigen encounter in SLOs.

In addition to antigen-specific BCR, which acts as a B cell intrinsic signal, Tfh-secreted cytokines function as B cell extrinsic signals that regulate B cell development within SLOs. One of the more well-studied cytokines is IL-21, which binds to the receptor IL-21R on the surface of T and B cells and induces activation of the transcriptional activator STAT3 (8, 41, 42). IL-21 can have opposing effects on B cell proliferation and differentiation depending on whether CD40 is also stimulated. Ligation of CD40 on B cells by CD40L on T cells synergizes with IL-21 to activate B cell proliferation; however, when CD40 remains unbound, IL-21 inhibits proliferation and promotes apoptosis (43). Pre-GC B cells require IL-21 to migrate from the follicular periphery to the center follicle, which is necessary for GC formation (44).

In conjunction with IL-21, IL-4 is required for proper GC development. Loss of IL-21 and/or IL-4 signaling results in small GCs *in vivo*, suggesting that these cytokines are an imperative form of Tfh cell-help for pre-GC B cells (44). Gonzalez and colleagues showed that IL-21 and IL-4 are not required to induce GC B cells, which are identified by BCL6 expression, at the T-B border. In the first three days of the adaptive immune response, loss of signaling through IL-21 and IL-4 does not alter the proliferation rate nor the population size of BCL6^hi^ pre-GC B cells. However, their further survival is impaired as indicated by elevated cell death rates and increased levels of the apoptotic marker, activated caspase-3. Interestingly, loss of signaling from only one of these cytokines does not increase activated caspase-3, suggesting that IL-21 or IL-4 alone is enough to promote survival in the transition from the T-B border to the GC. Overall, these data suggest that signaling through IL-21 and IL-4 is not required to induce the GC response, but is required to maintain pre-GC B cells.

While mature B cell developmental pathways usually proceed through a combination of EF and GC pathways in response to infection, some pathogens primarily induce an EF response with a delayed GC response (18). The bacterium *Ehrlichia muris* (*E. muris*) suppresses splenic GC formation while *Borrelia burgdorferi* (B. burgdorferi) delays GC formation and promotes the production of EF, IgM expressing B cells in lymph nodes (45, 46). Additionally, *Salmonella enterica* typhimurium (*S*Tm) induces an early EF response while delaying GC formation for one month (19, 45, 47, 48). During *S*Tm infections in mice, this delay in GC formation likely results from high levels of IL-12, which prevents Tfh differentiation by upregulating T-bet, a transcription factor that directs T cells to a helper type 1 (Th1) fate (18, 49). The resulting deficiency in Tfh development skews B cells toward an early EF response (50). Whether late GC formation occurs due to repopulation of Tfh cells remains uncertain as the numbers of Tfh cells were not analyzed past 17 days post-infection (50). Exactly why and how these bacteria induce EF responses while delaying or inhibiting GC responses remains unclear and suggests that these pathogens could be a useful infection model to evaluate the development of an EF response.

## Extrafollicular B Cell Development

The EF response provides the first wave of humoral protection by producing antibody secreting cells (ASCs) and MBCs as early as 3 days after antigen encounter (19). During the EF response, activated B cells migrate to the bridging channels of the spleen and the medullary cords of the lymph nodes, primarily due to their aforementioned expression of CXCR4 and EBI2 (20, 35, 51, 52). There, the B cells receive proliferation and survival signals such as IL-6 and a proliferation-inducing ligand (APRIL) from dendritic cells (DCs) and macrophages (51, 53). These signals cause the B cells to rapidly divide and form extrafollicular foci, where EF ASCs are generated (51, 53).

Although the majority of EF-derived Igs are IgM, activated EF B cells can undergo CSR to produce IgG and IgA (54, 55). During the EF response to certain T-independent antigens, stimulation of the BCR synergizes with toll-like receptors (TLRs) to induce CSR (56). Both BCR and TLR signaling induce NF-κB, a transcription factor required for AID expression (56, 57). The T-independent antigen lipopolysaccharide (LPS) has been proposed to stimulate both of these receptors by activating the BCR through its repetitive polysaccharide moiety as well as TLR4 through its lipid A moiety (56). Blocking CD79, a BCR co-receptor, *via* α-CD79 antibody inhibits BCR signaling and severely reduces CSR to IgG1 upon stimulation with LPS and IL-4, suggesting that signaling by BCR and TLR4 is required for CSR following LPS treatment (56, 58).

In the EF response to T-dependent antigens, activation of both the BCR and CD40 initiates strong phosphatidyinosital-3 kinase (PI3K) signaling that augments proliferation of activated B cells (59). However, strong PI3K signaling also inhibits CSR (59, 60). In T-dependent EF responses, antagonism of the PI3K signaling pathway *via* activity of PI3K interacting protein 1 (PIK3IP1) promotes CSR, which was recently demonstrated by Ottens and colleagues (61). Mice with CD19-cre mediated PIK3IP1 deletion show delayed production of IgG1 after immunization with NP conjugated to keyhole limpet hemocyanin (KLH), a T-dependent antigen. Interestingly, class switching in GC B cells remained functional, suggesting a role for PIK3IP1 in CSR specifically within the EF T cell-dependent response. However, immunization of these mice with NP-Ficoll, a T-independent antigen, did not delay IgG1 production, indicating that the T-independent EF response was not impaired by loss of PIK3IP1. These data suggest that PIK3IP1 is required to limit the high levels of PI3K signaling that results from the combination of CD40 and BCR stimulation in T-dependent EF responses, permitting CSR. Additional studies examining how PI3K regulates CSR in T-dependent EF responses will provide insight into the activation and development of mature B cells. This could be tested by overexpressing PI3K in B cells exposed to NP-Ficoll and evaluating the levels of isotype-switched B cells in the presence and absence of PI3KIP1. In the absence of CD40 stimulation, this model could reveal whether PI3K antagonizes CSR in a T-independent response.

Although they undergo CSR, EF B cells typically have not undergone SHM, which occurs in the GC dark zone (62). However, recent studies indicate that *E. muris* and *S*Tm infections, which elicit an EF response without typical GC formation, can also initiate SHM at very low levels (48, 63). High-throughput sequencing of mRNA of B cells and PBs from microdissected EF foci of mice infected with *E. muris* and *S*Tm revealed low levels of mutations in V regions, suggesting SHM occurs in these cells (48, 63). However, currently no evidence supports SHM occurring outside of the GC in humoral responses that develop classically described GC responses. If EF SHM only occurs when GC formation is delayed or does not occur, it could represent a desperate attempt by the immune system to produce high affinity antibodies, which normally form in the GC (3, 31). Investigation into the specific cytokines and chemokines secreted in response to *E. muris* and *S*Tm infections may elucidate what specific factors allow and promote EF SHM.

Regardless of SHM status, PBs produced from the EF response expand rapidly and secrete antigen-specific antibodies (64). PB differentiation during the EF response requires the same signals and transcriptional program as PB development in the GC: strong BCR signaling induces the expression of interferon regulatory factor 4 (IRF4), which in turn activates *Prdm1* whose protein product, BLIMP-1, is essential for PB development (40, 65, 66). BLIMP-1 suppresses genes involved in GC B cell and MBC formation and upregulates genes associated with the plasma cell fate (16, 67). Although the developmental program for PBs are the same in the EF response and the GC, Igs produced by EF PBs generally exhibit relatively low antigen affinity as compared to those stemming from the GC reaction, which undergo affinity maturation (68).

BCR affinity determines EF PB differentiation and fate (11, 69). Activated mature B cells with high affinity BCRs preferentially differentiate into EF PBs and among these EF PBs, those with higher affinity BCRs proliferate at a faster rate than those with lower affinity BCRs (11, 14). Interestingly, recent evidence indicates that hyperactive BCR signaling is disadvantageous to EF PB formation (69). To study the role of hyperactive BCR signaling during EF PB development, Yam-Puc and colleagues conditionally inactivated SH2 domain–containing phosphatase-1 (SHP-1), an antagonist of BCR signaling, in mice using a Cγ1-cre. Increased levels of SYK phosphorylation in SHP-1-deleted splenic B cells indicated enhanced levels of BCR signaling. Because strong BCR signaling promotes EF PB differentiation (14), these mice with heightened BCR signaling were expected to show increased numbers of EF PBs upon immunization with sheep red blood cell (SRBC). Surprisingly, these mice had smaller EF foci and higher levels of apoptotic EF PBs than wild-type (WT) controls, suggesting a maximal limit to BCR signaling for PB development. However, as SHP-1 is a phosphatase with several targets, SHP-1 could promote EF PB survival through pathways independent of BCR signaling (70). Further studies using alternative models for BCR hyperactivity, such as constitutive activation of BCR signal transducer SYK, should be performed to confirm these findings. The exact threshold of BCR signaling needed for EF PB differentiation and expansion could also be examined, potentially by injecting α-BCR or antigens of varying affinity.

In addition to PBs, low affinity MBCs can also be produced before GC formation; however, whether these MBCs are formed within the B cell follicle before the formation of GCs or in the extrafollicular region is unclear (71). While the developmental pathways from which GC-independent MBCs arise are not yet fully understood, these MBCs develop directly from antigen-activated mature B cells and do not require BCL6 or IL-21, both of which are required for GC MBC formation (72–74). Recent evidence indicates that GC-independent MBC development relies on B-cell activating factor receptor (BAFF-R) (75). BAFF-R promotes the survival of mature, naive B cells after binding the B-cell activating factor (BAFF) ligand that is expressed by DCs, follicular DCs (FDCs), and macrophages (76). Using mice that express BCRs against HEL (SW_HEL_) with germline deletions for BAFF-R, Lau and colleagues demonstrated that these mice had drastically reduced percentages of IgG1^+^ MBCs with unmutated IgH variable domains and increased percentages of IgG1^+^ MBCs with mutated IgH variable domains following immunization with HEL conjugated to SRBC (75). Conversely, overexpression of BAFF-R by retroviral transduction in SW_HEL_ B cells, which were subsequently transferred into WT mice, significantly expanded the percentage of unmutated IgG1^+^ MBCs. The population of unmutated IgG1^+^ MBCs was interpreted to be GC-independent due to a lack of SHM and the absence of the Y53D mutation in the Ig heavy chain variable region, which is frequently observed in SW_HEL_ GC affinity maturation, and thus implicating BAFF-R in GC-independent MBC development. In a complementary experiment, mice were administered bromodeoxyuridine (BrdU)-containing water and the B cells positive for both BrdU and the MBC marker CD38 were analyzed. Highly replicating cells, as marked by low levels of BrdU, were identified as MBCs stemming from the GC, while MBCs that differentiated before the GC reaction were identified with high levels of BrdU. WT mice treated with a BAFF-neutralizing antibody showed a large decrease in IgM^+^BrdU^+^CD38^+^ and IgG1^+^BrdU^+^CD38^+^ B cells, accompanied by a small but significant decrease in both affinity-maturated BrdU^-^ MBCs and overall GC B cells 14-days post-treatment. Together, these data indicate that BAFF-R is necessary for GC-independent MBC development but dispensable for MBCs stemming from the GC. However, some of the unmutated MBCs and BrdU^+^CD38^+^ MBCs analyzed in these studies did arise from the germinal center, and additional model systems that permit accurate identification of MBC precursors both in and outside of the GC could identify GC-independent MBC populations.

## Germinal Center B Cell Development

### Germinal Center Formation and Maintenance

**Figure 2:**
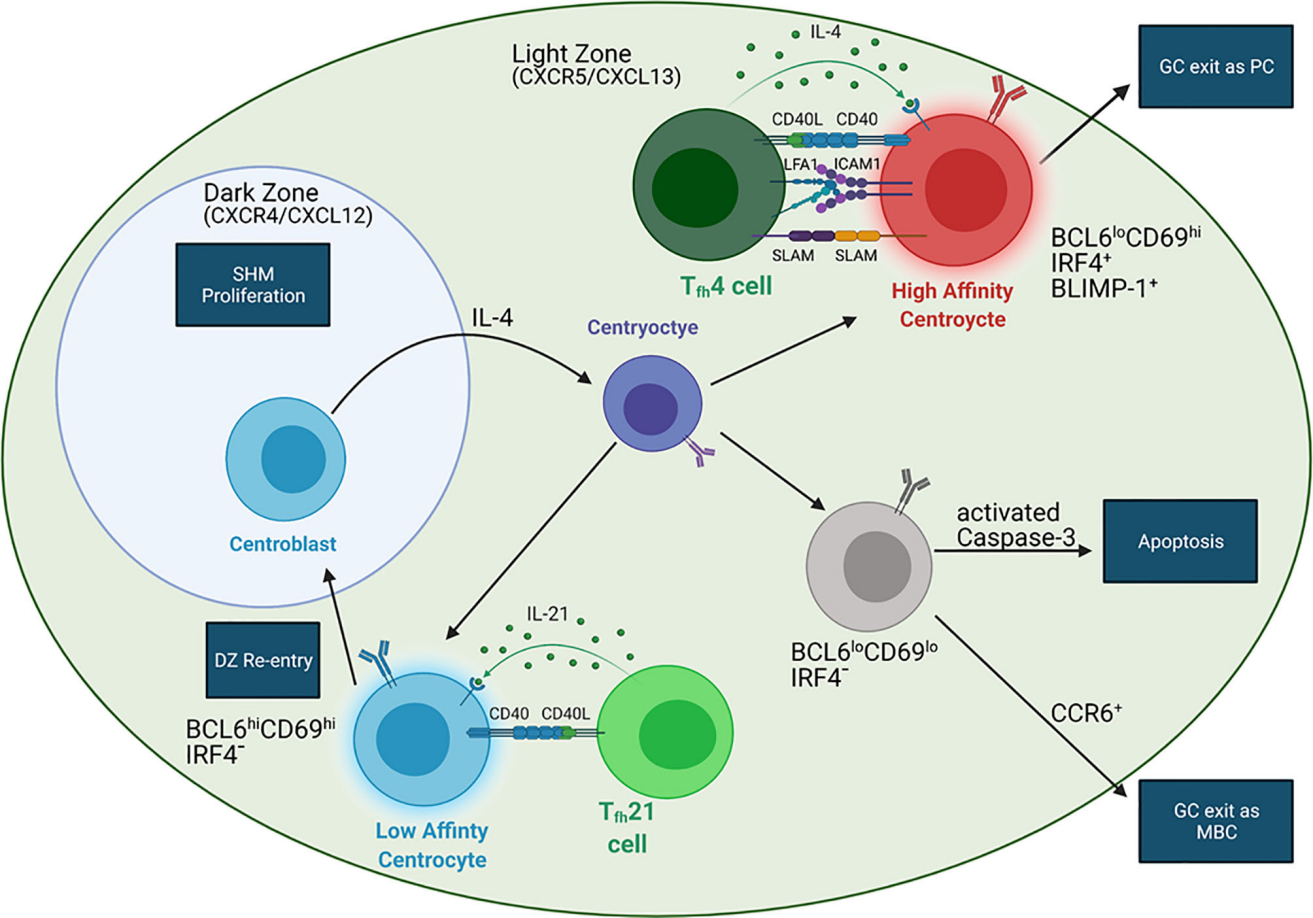
BCR affinity controls the fate of GC B cells. In the LZ, B cells with high affinity BCRs (red) interact with IL-4 producing Tfh4 cells (dark green) distal to the DZ. IL-4 can induce BLIMP-1 expression in these B cells, leading to GC exit and formation of long-lived plasma cells (LLPCs). These B cells have stronger interactions with Tfh B cells than B cells expressing low affinity BCRs due to upregulation of ICAM1 and SLAM. LZ B cells with low affinity BCRs (blue) interact with IL-21 secreting Tfh21 cells (light green) more proximal to the DZ. These B cells do not upregulate BLIMP-1 but are converted into centroblasts (blue cell in DZ) *via* IL-21, allowing for DZ entry and GC recycling. Centroblasts do not express a functional BCR. Migration into the DZ is dependent on CXCR4, which responds to the DZ chemokine CXCL12. When the centroblasts exit the DZ, they convert into centrocytes with varying BCR affinities (purple) *via* IL-4 signaling. The affinity of the newly mutated BCR (purple) may or may be increased, which will influence the next step in B cell development. Migration into the LZ depends on CXCR5, which responds to the LZ chemokine CXCL13. Another population of LZ B cells (gray) are relatively quiescent and may be either MBC precursors or an apoptotic population. CCR6 expression indicates MBC precursors while activated caspase-3 expression indicates apoptotic B cells. Additionally, CD40-CD40L interactions are required in all Tfh-B interactions.

The GC response provides another pathway for B cell differentiation in response to antigens. After initial activation by cognate antigens, B cells fated for the GC reaction migrate to the center of the follicle and rapidly divide, beginning the formation of the GC (77). Initiation of the GC reaction requires B cell co-stimulation by ligands expressed on the surface of T cells and APCs (78). CD40 stimulation is imperative for GC formation as CD40-deficient mice exhibit defective GC formation in both T-dependent and T-independent responses (79, 80). Within the GC, Tfh-derived cytokines and chemokines regulate mature B cell development (**Figure 2**) (31, 81). B cells in turn modify their responsiveness to these signals by modulating the expression of chemokine and cytokine receptors, which is influenced by BCR signaling and interactions with Tfh cells (12, 20, 34, 82, 83).

GC formation also requires expression of the transcriptional repressor BCL6 (25, 84, 85). BCL6 is significantly upregulated in GC B cells and Tfh cells and is considered the master regulator of the GC (10, 85, 86). Loss of BCL6 in mice impairs GC formation but permits the EF response, suggesting its function is limited to the GC response (87, 88). BCL6 suppresses transcription of p53 and p21 to inhibit apoptosis and cell-cycle arrest (89, 90). This allows for the rapid proliferation of B cells that is required for GC formation and the induction of genetically programmed DNA mutations necessary for SHM (78). BCL6 also retains B cells within the GC and prevents PC differentiation by inhibiting the expression of BLIMP-1 (78, 91). Additionally, BCL6 inhibits expression of PDL1 in GC B cells to maintain the necessary Tfh population (92).

BCL6 expression in GC B cells is both promoted and inhibited by BCR signaling and CD40 stimulation (93). Strong signaling induced by high affinity BCRs and CD40 stimulation *via* membrane-bound CD40L on the Tfh cell surface leads to mitogen activated protein kinase (MAPK)-dependent phosphorylation of BCL6 and subsequent degradation of BCL6 through the ubiquitin-proteosome pathway (93–95). Specifically, the MAPK, extracellular signal-regulated kinase 1 and 2 (ERK1/2), signals BCL6 for degradation. Conversely, p38, which is also a MAPK activated by BCR signaling and CD40 stimulation, promotes BCL6 expression. Interestingly, soluble CD40L, compared to membrane-bound, activates p38 without activating ERK1/2, permitting BCL6 expression (93). This suggests that the mode of stimulation is also an important factor in mediating the GC response through BCL6. Furthermore, while strong BCR signaling leads to BCL6 degradation, basal or tonic BCR signaling permits BCL6 expression to retain B cells in the GC. In this context, BCL6 inhibits expression of the apoptosis-promoting phosphatase PTPROt and permits survival of low-affinity B cells in the GC (94).

While BCL6 is considered the master regulator of the GC reaction, other proteins control GC B cell development. Like BCL6, BACH2 inhibits expression of BLIMP-1 and p21, thereby preventing premature PC differentiation and apoptosis, respectively (96–99). Furthermore, the inhibition of BLIMP-1 by BACH2 increases class-switched PCs, as mouse splenic B cells more readily become IgM-expressing PCs in the absence of BACH2 (98). Loss of BLIMP-1 on a BACH2^-/-^ background rescues the CSR deficiency of BACH2 single mutants, suggesting that inhibition of BLIMP-1 by BACH2 promotes CSR (98). Complete ablation of BACH2 following GC formation collapses the GC B cell population *in vivo* and increases the rate of B cell apoptosis *in vitro*, suggesting that BACH2 maintains GC B cell survival (96, 97). The loss of GC B cells in BACH2^-/-^ mice may also be due to the role of BACH2 in BCR-induced B cell proliferation, which is significantly reduced in response to α-IgM stimulation because BACH2^-/-^ B cells are unable to transition into S phase (97). Interestingly, BACH2^-/-^ B cell proliferation in response to LPS is comparable to WT B cells, suggesting that BACH2 regulates a BCR-specific proliferation pathway (97). Thus, in GC B cells BACH2 promotes progression through the cell cycle and inhibition of apoptosis upon BCR stimulation (97). Additional experiments evaluating the temporal regulation of BACH2 expression could improve our understanding of its role in GC B cell maintenance and clonal expansion.

Inhibition of p21 transcription by BACH2 and BCL6 is imperative to the GC reaction; however, repression of p21 can also be carried out *via* epigenetic modifications (90, 97, 100). The methyltransferase EZH2 binds to the *CDKN1A* locus, which encodes for p21, and induces H3K27me3 to repress transcription. Similar to BCL6 and BACH2, EZH2 is required for GC formation (100). To repress *CDKN1A* transcription, EZH2 directly binds the *CDKN1A* promoter while BCL6 interacts with the transcriptional activator MIZ-1, which also binds to the *CDKN1A* promoter, indicating complementary modes of repressing p21 expression by BCL6 and EZH2 (90, 100). On the other hand, BACH2 binds upstream of the *CDKN1A* promoter, suggesting it may work in tandem with EZH2 and/or BCL6 (97). Similar to BACH2 deficiency, loss of EZH2 suppresses the G1/S transition, providing further support for their synergistic activity in the regulation of p21 expression and cell cycle progression (100). Whether these proteins bind directly or indirectly to one another at the *CDKN1A* locus or whether they function in tandem or complementary genetic pathways requires further evaluation.

### Germinal Center Light and Dark Zones

Formation of the GC from the rapidly dividing B cells polarizes it into two zones, the dark zone (DZ) and light zone (LZ), which appear histologically distinct due to differing lymphocyte densities (62). Devoid of Tfh cells, the DZ contains B cells and FDCs and is the site of SHM (62). In contrast, the LZ contains Tfh cells, B cells, and FDCs and is the location of T-dependent selection of antigen-specific B cells (34, 62). DZ B cells, also called centroblasts, are highly proliferative and generally larger than LZ B cells, termed centrocytes (62). Centrocytes express mutated, functional BCRs whereas centroblasts only express non-functional BCRs, reflecting the aforementioned selection that occurs in the LZ and affinity maturation within the DZ **(** **Figure 2** **)** (34, 62).

The original model for GC entry proposed that B cells first enter the DZ, due to their expression of CXCR4, which is attracted to CXCL12 that is more abundant in the DZ than the LZ (101). Within the DZ, the cells undergo SHM and proliferate, and then downregulate CXCR4, while maintaining CXCR5 expression, permitting migration to the LZ where the ligand CXCL13 is expressed by FDCs (77, 101). Once in the LZ, the B cells stop dividing and undergo selection (62, 78). However, recent studies suggest a more dynamic model in which the DZ and LZ are less discrete compartments that allow GC B cells to cycle between the two zones (34). Two-photon laser microscopy studies in mice have revealed bidirectional trafficking of antigen-specific B cells between the DZ and LZ (102, 103). Additionally, dividing cells are detected in both the LZ and DZ, contradicting the idea that GC B cells exclusively proliferate within the DZ (102, 104). However, another study, using multiphoton microscopy and flow cytometry, shows that B cells only divide within the DZ (105). Victora and colleagues revealed a net movement of B cells from the DZ to the LZ and that Tfh cell help dictates whether a B cell will return to the DZ, largely supporting earlier models of B cell dynamics. Accordingly, the exact developmental and migratory paths of a GC B cell remain debatable, though they are most likely more dynamic than the original model.

The LZ is classically thought to be the site of CSR. This was first postulated by a study showing that centrocytes only express limited Ig isotypes, suggesting that isotype switching is initiated within GCs and after SHM (106). Additionally, 5′Sγ–Sμ3′ excision circles are detectable within GCs in human tonsils, suggesting that their deletion during CSR occurred in GC B cells (106). However, a recent study by Roco and colleagues refutes this assumption and postulates that CSR occurs before GC formation (107). Germline transcripts (GLTs), an indicator for the onset of CSR, and class-switched antibodies emerge 1.5-2.5 days post-immunization, whereas EFPBs and nascent GCs do not appear until 3.5 days post-immunization, suggesting that CSR occurs before GC formation. Additionally, expression of transcription factors and enzymes that regulate CSR, such as Foxo1, c-Myc and APE1, are downregulated in GC B cells. Moreover, both LZ and DZ B cells have markedly reduced GLTs, as compared to EFPBs and pre-GC B cells. However, this new model remains controversial, in part because a mechanistic understanding of the distinct factors that regulate, and consequently mark, AID activity specifically at V genes for SHM in GC B cells versus S regions during CSR in EFPBs remains elusive (18). In addition, as previously mentioned, CSR occurs in the EF response which raises the question of whether CSR is a GC-independent process or whether two distinct, CSR pathways exist: one specific to the EF response and one specific to the GC (54, 55).

In support of the hypothesis that CSR is a GC-independent process, Sundling et al. recently proposed that the increase in IgG^+^ B cells compared to IgM^+^ B cells in the GC results from stronger positive selection of IgG^+^ B cells and counter-selection of IgM^+^ cells (108). To eliminate ongoing CSR as the explanation for the increase in IgG^+^ and decrease in IgM^+^ GC B cells over time, Sundling and colleagues co-transferred MD4 and SW_HEL_ B cells into WT recipient mice. While both cell types are high affinity for HEL, MD4 cannot undergo CSR. After immunization with HEL, both MD4 and SW_HEL_ B cells expressing IgM in the GC decrease over time with the same kinetics, suggesting that the decrease in IgM^+^ GC B cells is not due to ongoing CSR. Similarly, co-transfer of SW_HEL_ B cells deleted for Sµ (ΔSµ), which cannot complete CSR, along with SW_HEL_ B cells WT for Sµ, yielded the same result. Furthermore, IgG1^+^ B cells spend more time in the cell cycle, more frequently enter the DZ, and are more likely to differentiate into PCs than their IgM+ counterparts, indicating stronger positive selection for IgG+ GC B cells (82, 108, 109). Along with the study by Roco et. al., these recent data support a model in which B cells undergo CSR at low levels prior to entering the GC, wherein high-affinity IgG^+^ GC B cells are positively selected over IgM^+^ and low-affinity GC B cells to undergo proliferation and differentiation into PCs (107, 108). Thus, the role of the GC is not to induce CSR, rather it is to expand the population of high affinity, class-switched B cells by inducing high rates of proliferation and differentiation into IgG^+^ PCs. However, this model for the role of the GC is not definitively accurate and requires further evaluation. Transfer of IgG^+^ SW_HEL_ B cells into SW_HEL_ AID knockout and WT mice could test this hypothesis by revealing if the IgG^+^ B cell population expands in comparison to the IgM^+^ population. Because AID-deficient B cells cannot undergo CSR, expansion of the IgG^+^ B cell population can be attributed to clonal expansion rather than ongoing CSR. If the model suggested by Sundling and colleagues is correct, then the transferred IgG^+^ SW_HEL_ B cells should outcompete the AID knockout B cells and unswitched WT B cells. If CSR does indeed occur in the GC, then in the WT mice, the endogenous (i.e. not transferred) B cells should switch and expand at a similar rate to the transferred, IgG^+^ B cells.

### Role of BCR Affinity in the Germinal Center

**Figure 3:**
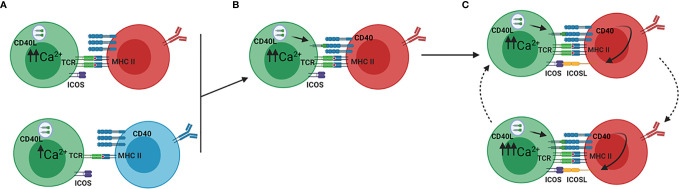
ICOSL driven feed forward model of serial entanglement. **(A)** Antigen specific B cells present antigen to Tfh cells *via* MHCII on the B cell surface. Tfh cells (green) bind the MHCII-antigen complex *via* the T cell receptor (TCR), which activates calcium signaling within the Tfh cell. B cells expressing high affinity BCRs (red) present more antigen than those expressing low affinity BCRs (blue), thereby inducing a more Ca^2+^ release. **(B)** Ca^2+^ signaling in the Tfh cells induces translocation of CD40L to the Tfh cell surface, which binds CD40 on the B cell surface. **(C)** Stimulation of CD40 on B cells induces the expression of ICOSL on the B cell. ICOSL binding to ICOS on the Tfh cell surface induces increased Ca2^+^ signaling, which again leads to translocation of CD40L to the Tfh cell surface. In this model, B cells increase their expression of ICOSL, which prepares them for stronger entanglement with Tfh cells. Adapted from reference (13).

The GC reaction is heavily influenced by BCR affinity and T-B interactions **(** **Figure 2** **)** (12, 13). High affinity LZ B cells have stronger interactions with GC Tfh cells as compared to low affinity LZ B cells because BCR signaling affects an ICOSL-dependent feed-forward mechanism of serial entanglement **(** **Figure 3** **)** (13). In this model, antigen presentation from MHCII on the B cell surface to Tfh cells activates the release of intracellular Ca^2+^ in Tfh cells, which induces CD40L localization to the Tfh cell surface (13, 110). B cells expressing higher affinity BCRs present more antigen than those expressing lower affinity BCRs, leading to more CD40L on the Tfh cell surface (13). Thus, higher affinity BCRs indirectly lead to stronger CD40 stimulation on the B cell through increased CD40L on the Tfh cell surface. CD40 stimulation leads to ICOSL expression on the B cell surface, which stimulates ICOS on the Tfh cell and increases intracellular Tfh Ca^2+^ signaling (13). Because Ca^2+^ signaling also induces the release of cytokines, such as IL-21 and IL-4, by Tfh cells, this model provides a mechanism by which higher affinity B cells receive more help from Tfh cells in the LZ (10, 111). This relationship between BCR affinity and ICOSL activity differs from interactions at the T-B border, where high affinity BCRs prevent ICOS-ICOSL interactions from inducing Tfh differentiation, as would be expected (20). Conversely, in the GC LZ, B cells expressing high affinity BCRs receive more help signals, through CD40 and cytokine signaling, by increasing Tfh activity through ICOS-ICOSL ligation (10, 13, 111).

Tfh-secreted cytokines IL-21 and IL-4 are critical for proper GC B cell development and migration within the GC **(** **Figure 2** **)** (10, 44, 111). In mice, inhibition of IL-4-signaling through the deletion of STAT6 results in an increased ratio of centroblasts:centrocytes (44). Conversely, deletion of the IL-21R results in a higher percentage of centrocytes (44). This suggests that IL-4 promotes conversion of centroblasts to centrocytes and IL-21 regulates development of centrocytes into centroblasts, which are localized to the DZ. Additionally, IL-21 maintains expression of BCL6 in GC B cells, while IL-4 plays a role in preventing apoptosis and, along with CD40 signaling, promotes isotype switching to IgG1 (10, 111).

While both IL-21 and IL-4 are secreted by Tfh cells in the GC LZ, an individual Tfh cell can only secrete one of these cytokines – Tfh4 cells produce IL-4, whereas Tfh21 cells produce IL-21 (83). Compared to Tfh21 cells, Tfh4 cells localize further from the DZ, closer to the periphery of the GC, and induce BLIMP-1 expression, suggesting they could be more involved in controlling PC differentiation and B cell exit from the GC (83). Conversely, Tfh21 cells induce BCL6 expression, which antagonizes BLIMP-1 and retains B cells in the GC (10, 83). Interestingly, a larger percentage of Tfh21 cells than Tfh4 cells appears within the first 8 days of the GC response, but between days 8 and 15 Tfh4 cells become the dominant population, suggesting that the role of IL-21 diminishes while the role of IL-4 increases as the GC reaction progresses (83). Based on the roles of Tfh21 and Tfh4 cells in the LZ, this could represent a change from affinity maturation to PC differentiation (83).

Along with these two populations of GC Tfh cells, the model of serial entanglement described earlier could provide a clearer understanding of LZ B cell development. Through increased ICOSL expression and antigen presentation on MHCII, B cells with high affinity BCRs induce increased Ca^2+^ signaling in Tfh cells, which will in turn secrete higher levels of IL-21 or IL-4 (**Figures 2**, **3**) (13).This suggests that these cytokines act as B cell extrinsic signals that direct B cell development based on BCR affinity, a B cell intrinsic characteristic (13). B cells that interact with Tfh4 cells at the LZ periphery may be induced to differentiate into IgG1-secreting PCs, if IL-4 signaling is strong enough (13, 83). If the BCR affinity is too low, then IL-4 signaling would not be sufficient to induce differentiation and these B cells will migrate toward the DZ, where they may interact with Tfh21 cells, transition into centroblasts, and enter the DZ to undergo affinity maturation (13, 44, 83).

Though Tfh cells are localized to the LZ, the strength of their interactions with LZ B cells influences the events in the DZ (82, 109). B cells that present higher amounts of antigen to Tfh cells in the LZ upregulate metabolism genes and drivers of cell cycle progression, such as c-Myc and E2F transcription factors, in the DZ (82). These B cells also show faster progression through S phase, faster replication fork progression, prolonged DZ retention, and increased rounds of replication (82, 109). These data suggest that increased Tfh cell help in the LZ influences the transcriptome of B cells that, in turn, controls their progression through and replication in the DZ. The data also suggest that events in the LZ can directly influence the events in the DZ and supports the idea that the DZ and LZ are not discrete compartments that act independently of each other (34). How Tfh cells communicate with B cells in the LZ to direct B cell development in the DZ remains to be determined. IL-21 could be an important LZ factor involved in controlling the transcriptome of DZ B cells as it activates c-Myc through the transcriptional regulator STAT3, promotes the centrocyte to centroblasts conversation, and is secreted by Tfh21 cells interacting with B cells proximal to the DZ (41, 42, 44, 83). However, IL-21 has a diverse range of effects on the B cell transcriptome, as represented by its ability to induce BLIMP-1 and BCL6, suggesting other signals are required (10, 41, 42, 111).

B cells expressing high affinity BCRs in the LZ preferentially differentiate into PCs while those expressing lower affinity BCRs preferentially remain in the GC for further affinity maturation (12). These subsets of LZ B cells can be identified by three LZ B cell markers: BCL6, CD69, and IRF4 **(** **Figure 2** **)** (12). CD69 marks positively selected LZ B cells after BCR or CD40 stimulation, IRF4 antagonizes BCL6 to promote BLIMP-1 expression and PC differentiation, and BCL6, as discussed previously, retains B cells in the GC (112–114). BCL6^lo^CD69^hi^IRF4^+^ LZ B cells are PC precursors that have stronger interactions with Tfh cells, as indicated by upregulation of ICAM1 and SLAM, in comparison to BCL6^hi^CD69^hi^IRF4^-^ LZ B cells that recycle through the GC for further affinity maturation (12). Consistent with this hypothesis, CD40 haploinsufficiency, which reduces Tfh-B cell interaction strength, significantly reduces the population size of PC precursors without affecting the overall GC B cell population or the GC recycling population (12). Future studies evaluating Tfh interactions with LZ B cells based on these three markers could provide insight into how Tfh cells regulate B cell development in the GC and improve our ability to track B cells through the GC. Because Tfh4 cells induce BLIMP-1 expression, the BCL6^lo^CD69^hi^IRF4^+^ PC precursors may be interacting with Tfh4 cells (12, 83). Conversely, the BCL6^hi^CD69^hi^IRF4^-^ GC recycling population may be interacting with Tfh21 cells near the DZ, as Tfh21 cells promote BCL6 expression (12, 83).

In addition to the two LZ B cell populations discussed, a third population has been identified as BCL6^lo^CD69^lo^IRF4^-^ (12). This gene expression profile reflects a quiescent population that is exiting the GC, suggesting it could be an MBC precursor population or an apoptotic population (12). Further studies examining CCR6, a GC marker for MBC precursors, and activated caspase-3 expression could help distinguish between these hypotheses **(** **Figure 2** **)** (115). Additionally, studies that identify the fate of the BCL6^lo^CD69^lo^IRF4^-^ LZ B cell population can be used to characterize PC precursors, GC recycling B cells, and MBC precursors. Interestingly, BACH2 haploinsufficiency inhibits MBC development and promotes PC differentiation (96, 98, 116). However, the role of BACH2 in MBC differentiation is independent of its effect on BLIMP-1 because deletion of both does not improve MBC differentiation relative to deletion of BACH2 alone (96). The mechanism by which BACH2 induces MBC differentiation in the GC remains unknown. Interestingly, BACH2 expression in GC B cells correlates inversely with the strength of T cell help and BCR affinity, which is consistent with the idea that GC-derived MBCs arise from lower affinity B cells (96, 117). This result emphasizes the inter-relationship between BCR affinity, T cell help, and terminal B cell differentiation (96).

## Discussion

The humoral immune response is a dynamic, complex process that is regulated by many signals and interactions between B cells and other cell types, especially CD4^+^ Tfh cells. Even though B cell development has been extensively studied, the regulatory mechanisms that control this process are still being explored with new models and genetic engineering tools such as CRISPR. Recent studies have elucidated a role for BCR affinity in controlling B cell development through EF and GC responses (12, 13, 20, 69, 82, 109). At the T-B border and within the GC, high affinity BCRs direct B cells to differentiate into ASCs, while low affinity BCRs direct B cells to enter or remain in the GC for affinity maturation (11, 12, 14, 15). While the processes governing the choice between affinity maturation and differentiation into ASCs have become clearer, the processes that control B cell differentiation into MBCs remain unclear. While MBCs seem to mainly arise from lower affinity B cells, some high affinity LZ B cells become MBCs, suggesting that BCR affinity is not the only signal determining MBC fate (115, 117). However, these signals are unknown and further exploration of MBC differentiation could promote the production of vaccines that confer effective long-term immunity.

Additionally, the processes that guide B cells to complete Ig maturation (SHM and CSR) in SLOs remain under investigation. The presence of class-switched EF Igs and recent molecular analysis of early CSR events suggests that CSR occurs outside of the GC, which challenges earlier models positing that CSR occurs within the GC (54, 55, 107). Similarly, SHM, which was previously thought to occur in the GC DZ, may also occur at low levels in EF B cells, particularly when GC formation is delayed or inhibited (48, 63). However, why EF SHM may occur during specific immune responses remains unknown. Improving our understanding of these processes and the locations in which they occur will provide us insight into Ig maturation and B cell development in SLOs and new model systems to produce effective therapeutic antibodies or vaccines.

## Author Contributions

AJW, TC-E, and MA wrote the manuscript. BQV edited the manuscript. All authors contributed to the article and approved the submitted version.

## Funding

This work was supported by The National Institute on Minority Health and Health Disparities (5G12MD007603), The National Cancer Institute (2U54CA132378), and The National Institute of General Medical Sciences (1SC1GM132035).

## Conflict of Interest

The authors declare that the research was conducted in the absence of any commercial or financial relationships that could be construed as a potential conflict of interest.

## Publisher’s Note

All claims expressed in this article are solely those of the authors and do not necessarily represent those of their affiliated organizations, or those of the publisher, the editors and the reviewers. Any product that may be evaluated in this article, or claim that may be made by its manufacturer, is not guaranteed or endorsed by the publisher.
